# Reply to Comment on Nakai, Y.; Makizako, H.; Kiyama, R.; Tomioka, K.; Taniguchi, Y.; Kubozono, T.; Takenaka, T.; Ohishi, M. Association between Chronic Pain and Physical Frailty in Community-Dwelling Older Adults. *Int. J. Environ. Res. Public Health* 2019, *16*, 1330, doi:10.3390/ijerph16081330

**DOI:** 10.3390/ijerph17010175

**Published:** 2019-12-25

**Authors:** Yuki Nakai, Hyuma Makizako, Ryoji Kiyama, Kazutoshi Tomioka, Yoshiaki Taniguchi, Takuro Kubozono, Toshihiro Takenaka, Mitsuru Ohishi

**Affiliations:** 1 Department of Physical Therapy, School of Health Sciences, Faculty of Medicine, Kagoshima University, Kagoshima 890-8544, Japan; nakai@health.nop.kagoshima-u.ac.jp (Y.N.); kiyama@health.nop.kagoshima-u.ac.jp (R.K.); 2 Graduate School of Health Sciences, Kagoshima University, Kagoshima 890-8544, Japan; reha_tommy@yahoo.co.jp (K.T.); p.taniguchi0601@gmail.com (Y.T.); 3 Department of Cardiovascular Medicine and Hypertension, Graduate School of Medical and Dental Sciences, Kagoshima University, Kagoshima 890-0075, Japan; kubozono@cepp.ne.jp (T.K.); ohishi@m2.kufm.kagoshima-u.ac.jp (M.O.); 4 Tarumizu Municipal Medical Center, Tarumizu Chuo Hospital, Kagoshima 891-2124, Japan; takenaka@tarumizumh.jp

Seven participants had a diagnosis of dementia; the number of excluded participants differed based on different exclusion processes, and there was missing data of one participant with a dementia diagnosis. That is, one participant was excluded in the missing data before being excluded by the dementia diagnosis. Certification for long-term care in Japan occurs at the disability level, not at the frailty level [1,2]. Therefore, in many Japanese cohort studies, certification for long-term care is an exclusion criterion [3]. Also, in this study, participants who were already certified for long-term care were excluded from the discussion of the frailty state. There can be multiple causes of chronic pain that cannot be identified in a single community-based cohort study [4]. Further, in the current study, we did not explore the use of painkillers and, thus far, no discussion has taken place in this regard [5]. This is an aspect we would like to consider in future studies.
